# Heterogeneity of white adipocytes in metabolic disease

**DOI:** 10.1097/MCO.0000000000000885

**Published:** 2023-01-11

**Authors:** Josh Bilson, Jaswinder K. Sethi, Christopher D. Byrne

**Affiliations:** a Human Development and Health, Faculty of Medicine, University of Southampton; b National Institute for Health and Care Research Southampton Biomedical Research Centre, University of Southampton and University Hospital Southampton National Health Service Foundation Trust; c Institute for Life Sciences, University of Southampton, Southampton, United Kingdom

**Keywords:** adiposity, metabolic disease, snRNA sequencing, white adipocyte

## Abstract

This review aims to discuss the most recent evidence identifying the presence of distinct white adipocyte subpopulations in white adipose tissue (WAT) and how these may be altered with increasing adiposity and/or metabolic disease. We conceptualize how changes in adipocyte subpopulations may contribute to alterations in WAT function and the development of metabolic diseases such as type 2 diabetes mellitus (T2DM), nonalcoholic fatty liver disease (NAFLD) and cardiovascular disease (CVD).

**Recent findings:**

Studies utilizing novel analytical approaches support the existence of distinct white adipocyte subpopulations in both human and murine WAT. Adipocyte subtypes are potentially functionally distinct and may have different roles in WAT function and obesity-associated metabolic diseases.

**Summary:**

The exploration of white adipocyte heterogeneity using novel analytical technologies, has unveiled a new layer of complexity in the study of WAT biology. Interrogation of potential functional differences between adipocyte subpopulations and their role in the function of different WAT depots is now needed. Through understanding the mechanisms regulating white adipocyte subtype development and potential pathophysiological consequences of changes in the presence of adipocyte subpopulations, studies could provide novel therapeutic targets for the treatment of T2DM, NAFLD, and CVD.

## INTRODUCTION

In addition to serving as a storage depot for energy substrates, white adipose tissue (WAT) is an endocrine organ that contributes significantly to whole-body energy metabolism and homeostasis. It is the excessive accumulation of WAT that defines obesity, and WAT dysfunction is thought to link obesity to a host of metabolic complications. At a population level, obesity is an independent risk factor for multiple metabolic diseases including type 2 diabetes mellitus (T2DM) [1], nonalcoholic fatty liver disease (NAFLD) [2], and cardiovascular disease (CVD) [3]. Obesity-associated alterations in WAT function are thought to reflect an impairment in WAT plasticity and are thought to contribute to the development and progression of metabolic diseases [4,5]. Adipocytes are the major cell type found within WAT; however, multiple other cell types including immune cells, mesenchymal progenitor/stem cells, and preadipocytes are also present and collectively form the stromal vascular fraction (SVF) of WAT (i.e. cell types within WAT that are not mature adipocytes). Furthermore, whilst not discussed here, the function and mass of another type of adipose tissue (brown adipose tissue), which is known to have an important role in maintaining body temperature during cold exposure, has also been suggested to contribute to obesity-associated metabolic diseases (as reviewed in [6]).

One approach that has been used to explore and identify changes in WAT function is via the exploration of whole-tissue gene expression profiles using RNA sequencing (RNAseq), which enables researchers to identify the presence and quantity of RNA in biological samples. Advances in RNAseq technologies over the last decade have facilitated the exploration of gene expression profiles at the level of single cells (scRNAseq). Such advances have consequently permitted the identification and further exploration of WAT SVF cell populations in both physiological and obesity-associated pathophysiological settings [7]. However, the exploration of white adipocyte (which herein will be referred to only as adipocytes) heterogeneity has only recently become possible and is rejuvenating the way we view this previously considered uniform cell type. Indeed, multiple adipocyte subpopulations have now been identified within both SAT and VAT. This builds on previous observations of the appearance of morphologically distinct adipocytes in WAT depots that also appear functionally intermediate between white and brown adipocytes (i.e. so-called ‘beige’ adipocytes) [8]. It is plausible that changes in the presence of adipocyte subpopulations and their function contribute to the known association between WAT dysfunction and metabolic disease. In this review, we focus on recent emerging evidence that demonstrates the existence of distinct adipocyte subpopulations within WAT in both humans and in murine models. We also highlight how specific adipocyte subtypes may be particularly relevant with increasing adiposity and metabolic diseases, such as T2DM, NAFLD, and CVD and provide some thoughts for future studies. 

**Box 1:**
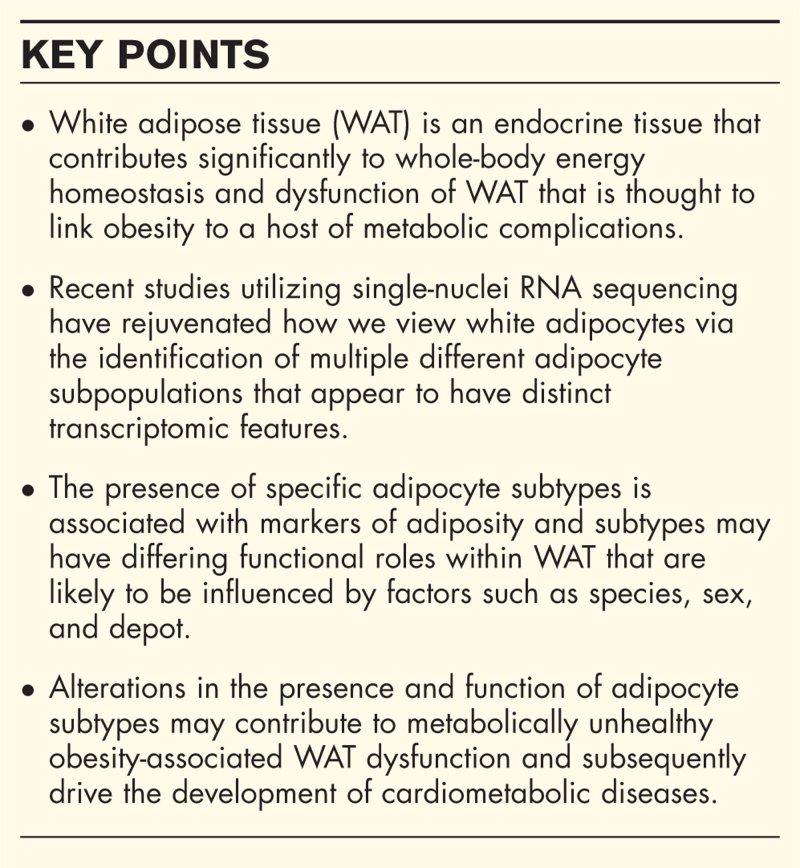
no caption available

## THE EXISTENCE OF WHITE ADIPOCYTE SUBPOPULATIONS

Earlier work demonstrated that subcutaneous adipose tissue (SAT) in both humans and mice is functionally distinct compared with visceral adipose tissue (VAT), and VAT is more closely associated with metabolic disease risk [9]. More recently, studies on human WAT using scRNAseq [10] and murine WAT using single-cell proteomics [11], have demonstrated depot-specific differences in WAT SVF cell populations that likely contribute to differences in tissue function and response to metabolic demand. The emergence and application of novel analytical techniques have facilitated the exploration of adipocyte populations at a single-cell level; something, which was previously unachievable because of the fragility of highly lipid-laden adipocytes making them incompatible with single-cell separation and sorting strategies [12^▪▪^]. In order to overcome this, rather than sequencing a whole cell, RNA within nuclei isolated from WAT can be sequenced via single-nuclei RNAseq (snRNAseq) consequently enabling the transcriptomic profiling of single adipocytes. The technical differences, advantages, and limitations of single-cell and snRNAseq for the study of WAT biology have been recently described in detail by Yang Loureiro *et al.*[13].

Historically, unilocular adipocytes were considered to be uniform in both form and function; however, we now appreciate that this somewhat simplistic view is likely inaccurate. Multiple snRNAseq studies have identified adipocyte subpopulations within murine and human WAT depots [12^▪▪^,^14^,^15^^▪▪^,16^▪^,17^▪^] (Table 1). In line with earlier findings identifying the presence of distinct adipocyte subpopulations in epididymal WAT from lean and obese male mice [14], human abdominal SAT was also found to predominantly consist of two distinct subclasses of adipocytes [15^▪▪^]. The first subclass referred to as Adipo^*LEP*^ (because of a high expression of the leptin-producing gene) was found to have an enriched expression for genes encoding proteins involved in cell–cell matricellular interactions (*TNS1* and *SPTBN1*) and modifiers of leptin signalling/secretion (*PTPN11* and *DDR2*) [15^▪▪^]. In contrast, the second subclass referred to as Adipo^*PLIN*^ (because of a high expression of lipid droplet proteins, perilipin-1, and perilipin-4) had an enriched expression of genes involved in glucose and lipid metabolism as well as adiponectin secretion [15^▪▪^]. Through implementing novel approaches that enabled the authors to identify the location of each adipocyte subclass within WAT samples, subclasses Adipo^*PLIN*^ and Adipo^*LEP*^ were found to colocalize whilst having a reciprocal expression pattern for genes involved in triglyceride (TAG) biosynthesis and hydrolysis (highly expressed in Adipo^*PLIN*^ and underrepresented in Adipo^LEP^). Thus, it is possible that anatomically related subpopulations of adipocytes may have functionally different roles in lipid handling within the same WAT depot [15^▪▪^]. This study also identified a third less abundant adipocyte subclass (Adipo^*SAA*^) characterized by a distinct expression of retinol-binding proteins. The authors speculate that Adipo^*SAA*^ may have a particular role in the modulation of WAT inflammation [15^▪▪^]. Additional studies are warranted to explore the role of adipocyte subtypes in the regulation of tissue inflammation in the context of obesity-associated WAT dysfunction.

**Table 1:**
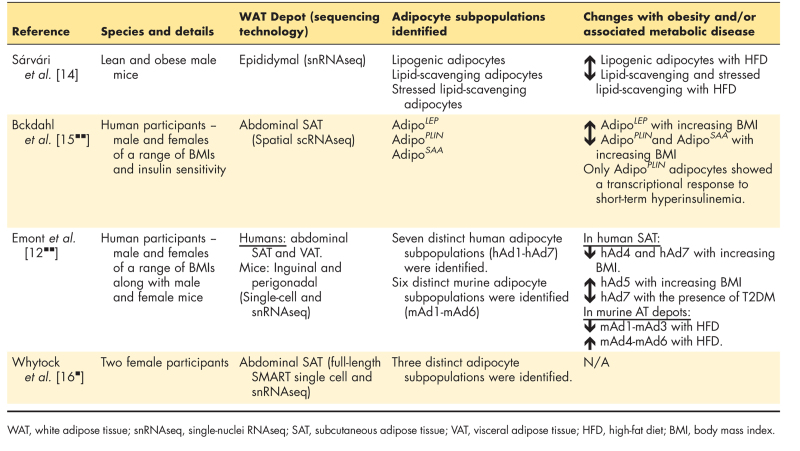
Recent studies identifying adipocyte subpopulations in human and/or murine white adipose tissue depots

Using snRNAseq, Emont *et al.*[12^▪▪^] detected seven distinct adipocyte subpopulations within paired human abdominal SAT and VAT biopsies and noted strong depot-specific associations of adipocyte subtypes. Compared with VAT, the presence of adipocyte subpopulations in SAT that were associated with a higher expression of genes involved in TAG biosynthesis, fatty acid desaturation, and lipogenesis [12^▪▪^]. Whilst yet to be explored, it is possible that such depot-specific differences in adipocyte subtypes are a contributing factor to the established depot-specific differences in lipid handling and metabolism. Interestingly, whilst some similarities in the types of adipocyte subpopulations in mouse models were observed, there was an absence of depot-specific subtype enrichment as seen in humans [12^▪▪^]. Whilst other SVF cell types may have a good cross-species concordance, the authors suggested that adipocytes in mice do not appear to map clearly to human adipocyte subpopulations [12^▪▪^]. Thus, caution should be exercised when attempting to extrapolate findings from studies exploring adipocyte subtypes in murine models to humans, at least until the clinical relevance of findings from preclinical models has also been confirmed in human tissues/cells.

A recent study has demonstrated that the quality of adipocyte transcriptomic profiling may be improved by using full-length snRNAseq, which results in an enhanced gene coverage that may permit more accurate profiling and identification of adipocyte subtypes within WAT [16^▪^]. This work also suggested that full-length snRNAseq of whole human SAT facilitated the identification of distinct adipocyte subpopulations whilst scRNAseq using isolated adipocytes from the same sample failed to identify any distinct subpopulations. This highlighted that the latter approach may not be appropriate for the identification of adipocyte subtypes [16^▪^]. It is also important to consider that studies using snRNAseq are marked with inherent transcript enrichment and detection bias as nuclear transcripts do not represent the total transcriptome of a single cell. This has recently been explored and a potential normalization strategy has been proposed with the aim of removing such technical detection biases [17^▪^].

## CHANGES IN WHITE ADIPOCYTE SUBPOPULATIONS WITH INCREASING ADIPOSITY AND IN METABOLIC DISEASE

We have highlighted evidence from snRNAseq studies supporting the existence of distinct adipocyte subtypes and potential depot-specific differences in the same individuals. However, are these subtypes altered with increasing adiposity and/or in obesity-associated metabolic diseases? Indeed, some evidence now suggests that shifts in the proportion of specific adipocyte subpopulations occur in parallel with increasing markers of adiposity and/or in the presence of metabolic disease (Table 1). In male mice, high-fat diet-induced obesity resulted in a shift from lipogenic to lipid-scavenging and stressed adipocytes with a particular downregulation of lipogenic genes in epidydimal WAT compared with lean (chow diet-fed) controls [14]. Integrative analyses of spatially resolved and scRNAseq SAT transcriptomic data from women indicated that the presence of adipocytes with a high expression of the gene-encoding leptin, *LEP* (Adipo^*LEP*^) was positively associated with BMI, whilst those with a high expression of adiponectin and genes involved in glucose and lipid metabolism (Adipo^*PLIN*^) were inversely associated with BMI [15^▪▪^]. This study also demonstrated that short-term exposure to insulin (following a hyperinsulinemic–euglycemic clamp) induced a robust transcriptional response specifically in Adipo^*PLIN*^, which also correlated with overall insulin sensitivity [15^▪▪^]. These findings could suggest that the capacity of WAT to respond to insulin is determined by the presence and function of a specific adipocyte subtype rather than the overall capacity of the depot to respond to insulin *per se*[15^▪▪^]. Furthermore, these findings may indicate that shifts in adipocyte subpopulations with increasing adiposity may modify the secretion of adipokines, which are known to be associated with metabolic diseases, such as T2DM, NAFLD, and CVD [18,19].

In WAT, adipocytes were found to be the cell type most likely to mediate the association between cellular changes in SAT with the presence of T2DM adjusted for BMI [12^▪▪^]. Specifically, only one adipocyte subtype (hAd7) in SAT was found to be associated with the presence of T2DM whilst only representing a small proportion (1%) of total adipocytes [12^▪▪^]. Despite the potential function of hAd7 being unclear, the presence of this subtype and its gene markers were found to be inversely associated with HOMA-IR, suggesting that changes in the presence of this subtype are linked to the development of T2DM and systemic insulin resistance [12^▪▪^]. Adipocytes were also found to be most strongly associated with BMI-adjusted waist-to-hip ratio (a measure of WAT distribution) supporting the notion that the adipocyte subtypes are likely to be depot-specific. Although not statistically significant, changes in human SAT adipocytes were also found to be associated with high-density lipoprotein (HDL) concentrations [12^▪▪^]. Similarly, the proportion of so-called ‘basal’ SAT adipocytes (hAd1) and changes in low-density lipoprotein (LDL) concentrations had a near-significant association and the expression of selective genes from this subtype (*NRCAM*, *PCDH7*, *PEMT*, and *VGLL3*) were positively associated with LDL concentrations in humans [12^▪▪^]. Such evidence could indicate that changes in the proportion and function of specific adipocyte subpopulations are associated with the development of a proatherogenic lipid profile; however, this is yet to be explored.

Despite the feasibility of using snRNAseq to interrogate adipocyte heterogeneity in WAT, it is important to consider that we do not yet understand the roles of different cell subpopulations on whole tissue function or on whole-body metabolic health. Exploring SVF WAT progenitor cell populations using scRNAseq, recent work has suggested that clustering in transcriptomic profiles was largely driven by the anatomical location of specific WAT depots (i.e. SAT vs. VAT) [20]. Conversely, clustering in proteomic profiles highlighted functional differences between cell subtypes suggesting that integration of both scRNAseq and proteomics may be required to identify functional differences between WAT cell subtypes [20]. Furthermore, the authors also report that the correlation between protein and corresponding transcript in WAT progenitor cell subgroups was influenced by both sex and depot [20]. Similarly, ageing in mice has been shown to associate with depot-specific emergence of adipose progenitor cells that appear to lose adipogenic capacity, suggesting that the cellular composition of WAT (and potentially adipocytes) dynamically changes with ageing [21]. However, whether or not similar observations extend to adipocytes, and/or whether adipocyte subtypes are altered with ageing and/or menopausal status is currently unknown and warrants further investigation. It is also unknown whether adipocyte subpopulations have differing susceptibility to cellular senescence (that aggravates WAT inflammation) and whether mechanisms accelerating adipocyte senescence, such as those recently identified by Lee and colleagues [22], impacts specific or all subpopulations. Furthermore, whilst efforts have been made to understand the difference in adipocyte subpopulations, currently, no information is available comparing these cells in SAT found within the upper (i.e. abdominal) and lower (i.e. gluteo-femoral) regions of the body. Similarly, whether adipocyte subpopulations are different between so-called ‘deep’ SAT and superficial or dermal SAT (the latter may be mistaken for ‘deep’ SAT during biopsy sampling, particularly in individuals with morbid obesity) has yet to be explored.

## FUTURE PERSPECTIVES AND CONCLUSION

**FIGURE 1:**
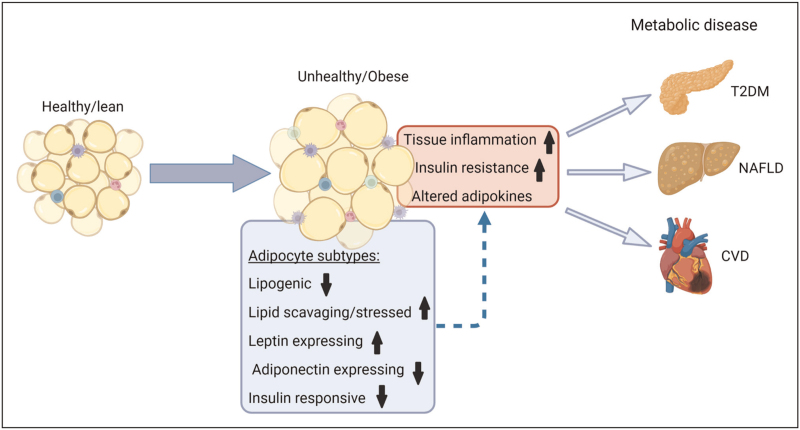
Obesity-associated alterations in adipocyte subpopulations may contribute to tissue dysfunction and an increased risk of cardiometabolic disease. Studies have suggested that obesity and/or metabolic dysfunctions such as T2DM and IR are associated with shifts in the proportions of specific adipocyte subpopulations. Such changes could be hypothesized to be a contributing factor behind the development of obesity-associated WAT dysfunction that typically manifests in increases AT inflammation, insulin resistance, and alterations in adipokines. Consequently, these changes may be a risk factor for the development of various cardiometabolic diseases including T2DM, NAFLD, and CVD. It is important to note that studies exploring changes in adipocyte subpopulations have predominantly explored SAT, more studies are required to confirm whether or not these changes are also observed in VAT. Figure was created using Biorender.com. CVD, cardiovascular disease; T2DM, type 2 diabetes mellitus; NAFLD, nonalcoholic fatty liver disease.

Whilst sparse, current evidence from both murine models and humans suggests that shifts in specific adipocyte subpopulations within WAT are more closely associated with changes in BMI and obesity-associated metabolic dysfunction. Figure 1 illustrates schematically how alterations in adipocyte subpopulations within WAT may contribute to tissue dysfunction and an increased risk of cardiometabolic disease. Reductions in body weight (via dietary and/or lifestyle modifications or glucagon-like peptide 1 agonism) is known to reverse/treat metabolically unhealthy obesity-associated WAT dysfunction and metabolic diseases, such as those shown in Fig. 1. However, it is not known whether weight loss impacts the distribution and presence of adipocyte subpopulations within WAT and whether these changes subsequently contribute to improvements in tissue function and are metabolically beneficial. Similarly, peroxisome proliferator-activated receptor (PPAR)-γ agonists, such as pioglitazone, are thought to improve cardiometabolic health via the promotion of WAT expansion (adipogenesis) within subcutaneous depots [23,24]. One recent randomized placebo-controlled trial suggested that the adipogenic effects of pioglitazone are more prominent in femoral (i.e. lower body) SAT compared with abdominal SAT in metabolically healthy obese women [24]. Whether PPAR-γ agonists such as pioglitazone influence the distribution of adipocyte subpopulations with an increased lipid-handling capacity and whether these effects are depot-specific is currently unknown. The findings highlighting alterations in adipocyte subpopulations should be taken in context with those demonstrating the vast changes in SVF cellular populations that have also been shown to occur with obesity and associated metabolic diseases (as reviewed by [7]). Interactions between adipocyte subtypes and dynamically changing SVF cell populations are likely to be depot-specific and influenced by sex and ageing and disease stage; creating a challenge when trying to explore WAT biology at a single-cell level. Furthermore, as recently mentioned [25], it may be possible that interconversion occurs from one adipocyte subpopulation to another. Whilst this may explain some of the previously described spatial relationships between subtypes [15^▪▪^], it potentially adds an additional layer of complexity to our understanding. Mechanistic studies exploring the transition of adipocyte subtypes will be crucial over the next few years. Our ability to understand the characteristics and functionality of WAT depots at a single-cell level (both spatially and temporally) is governed by the existence and availability of appropriate methodology. This area is in its infancy and much work is needed to enable us to understand if, how, where and when changes in adipocyte subpopulations contribute to functional changes in a given WAT depot. Technologies, such as ‘Live-seq’ may eventually facilitate the temporal transcriptomic recording of single adipocytes [26]. Studies are now required to explore the relevance and role of specific adipocyte subtypes in the context of cardiometabolic diseases. It will be important for such studies to consider differences in WAT depots that may exist along with differences between sexes and with ageing. Developments in this area have the potential to drive forward novel therapeutic strategies for the targeted management of obesity-associated metabolic complications.

## Acknowledgements

*None.*

### Financial support and sponsorship

*J.B., J.K.S., and C.D.B. are supported by the National Institute for Health and Care Research through the NIHR Southampton Biomedical Research Centre (Grant Number IS-BRC-20004). This research was funded in part by the Wellcome Trust (Grant number 206453/Z/17/Z to J.K.S.).*

### Conflicts of interest

*There are no conflicts of interest.*

## REFERENCES AND RECOMMENDED READING

Papers of particular interest, published within the annual period of review, have been highlighted as:

▪ of special interest
▪▪ of outstanding interest
